# Rethinking social disconnection: does it always have a negative impact?

**DOI:** 10.1093/geroni/igaf122.3118

**Published:** 2025-12-31

**Authors:** Hyojin Choi, Robert Gramling, Maija Reblin

**Affiliations:** University of Vermont, Burlington, Vermont, United States; University of Vermont, Burlington, Vermont, United States; University of Vermont, Burlington, Vermont, United States

## Abstract

Social disconnection, which includes social isolation and loneliness, can negatively impact health and well-being in older adults. However, in reality, these relationships among social isolation, loneliness and well-being are complex. For example, isolated older adults might still feel lonely, but are satisfied with their situation, while those with numerous social connections may still experience loneliness. This study aimed to examine distinct subtypes of social disconnection based on the combination of loneliness and social isolation, and assess their impact on well-being. We analyzed data for all community-dwelling adults (n = 2,542) who participated in both the 2021 and 2022 samples of the National Health and Aging Trends Study. We found that 65% of older adults were neither isolated nor lonely (connected), 14% were only isolated, 15% were lonely but not isolated (lonely in a crowd), and 6% were both isolated and lonely. After controlling for demographic and socio-economic status, regression models show that, compared to the connected group, those who were disconnected or lonely in a crowd were likely to have more depression symptoms and negative emotions in the following wave. However, those who were only isolated did not show a significant difference in depression compared to the connected group. Compared to the connected group, all three other groups were more likely to report fewer positive emotions and more negative attitudes toward life. The findings suggest that feeling lonely is detrimental to well-being while social isolation alone may not be so. It highlights the need for community-based efforts to reduce loneliness.

